# Unilateral or bilateral vagotomy induces ovulation in both ovaries of rats with polycystic ovarian syndrome

**DOI:** 10.1186/1477-7827-11-68

**Published:** 2013-07-17

**Authors:** Rosa Linares, Denisse Hernández, Carolina Morán, Roberto Chavira, Mario Cárdenas, Roberto Domínguez, Leticia Morales-Ledesma

**Affiliations:** 1 Biology of Reproduction Research Unit, Physiology of Reproduction Laboratory, Facultad de Estudios Superiores Zaragoza, UNAM AP 9-020, CP 15000, México, DF, México; 2 Departament of Biology and Toxicology of Reproduction, Science Institute, Benemérita Universidad Autónoma de Puebla, Puebla, México CP 72000; 3 Instituto Nacional de Ciencias Médicas y Nutrición “Salvador Zubirán”, México, DF, México

**Keywords:** Polycystic ovarian syndrome (PCOS), Vagus nerve, Ovarian innervation, Ovulation, Vagotomy

## Abstract

**Background:**

Injecting estradiol valerate (EV) to pre-pubertal or adult female rat results in effects similar to those observed in women with polycystic ovarian syndrome (PCOS). One of the mechanisms involved in PCOS development is the hyperactivity of the sympathetic nervous system. In EV-induced PCOS rats, the unilateral sectioning of the superior ovarian nerve (SON) restores ovulation of the innervated ovary. This suggests that, in addition to the sympathetic innervation, other neural mechanisms are involved in the development/maintenance of PCOS. The aims of present study were analyze if the vagus nerve is one of the neural pathways participating in PCOS development.

**Methods:**

Ten-day old rats were injected with EV dissolved in corn oil. At 24-days of age sham-surgery, unilateral, or bilateral sectioning of the vagus nerve (vagotomy) was performed on these rats. The animals were sacrificed at 90–92 days of age, when they presented vaginal estrous preceded by a pro-estrus smear.

**Results:**

In EV-induced PCOS rats, unilateral or bilateral vagotomy restored ovulation in both ovaries. Follicle-stimulating hormone (FSH) levels in PCOS rats with unilateral or bilateral vagotomy were lower than in control rats.

**Conclusions:**

This result suggests that in EV-induced PCOS rats the vagus nerve is a neural pathway participating in maintaining PCOS. The vagus nerve innervates the ovaries directly and indirectly through its synapsis in the celiac-superior-mesenteric ganglion, where the somas of neurons originating in the SON are located. Then, it is possible that vagotomy effects in EV-induced PCOS rats may be explained as a lack of communication between the central nervous system and the ovaries.

## Background

Polycystic ovary syndrome (PCOS) is considered the most common cause of infertility in woman, with approximately 10 percent of women of reproductive age being affected with PCOS [1]. PCOS is characterized by a complex pathophysiology that includes anovulation, oligomenorrhea, follicular cysts, hyper-androgenism, hyper-estrogenism and variable levels of gonadotropins in blood [2-4]. PCOS diagnosis is based on the presence of three main features: the presence of more than 12 cysts in the ovaries, anovulation, and hyper-androgenism [5,6]. In some cases PCOS results in glucose metabolic disorders, cardiovascular diseases, dyslipidemia and cancer [4].

The etiology of PCOS is multifactorial and is attributed to genetic factors as well as primary defects of the hypothalamic-pituitary unit, micro-enviroment of the ovary such defects of intraovarian molecules involved in paracrine/autocrine regulation, such as insulin-like growth factor-I, and an overactive adrenal gland [4,7].

Ovarian functions are regulated by hormonal and neural signals. Hormonal signals regulating ovarian functions arise from the pituitary, adrenal, ovaries, thymus and thyroid; while neural signals arrive to the ovaries through the superior ovarian nerve (SON), the plexus ovarian nerve (OPN) and the vagus nerve. Neural signals modulate the effects of hormonal signals on the follicular, luteal and interstitial compartments [8-10].

Experimental models proposed to study the POCS include injecting estradiol valerate (EV), neonatal androgenization, the exposure of animals to constant light, and chronic stress induced by cold [11-13].

EV is a long acting estrogen. Injecting 2 mg of EV to infantile or adult rats results in the interruption of estrous cycle, persistent vaginal cornification, anovulation, formation of follicular cysts, alterations to the basal and pulsatile concentration of follicle-stimulating hormone (FSH) and luteinizing hormone (LH), as well as high concentrations of estradiol (E_2_) and testosterone (T) [14-16]. These effects are similar to those observed in women with PCOS.

Injecting luteinizing hormone-releasing hormone (LHRH) to EV-induced PCOS adult rat increased LH levels resulting in spontaneous ovulation [17,18]. There week after surgery unilateral ovariectomy to EV-induced PCOS adult rats, the remaining ovary showed follicles at all stages of development, corpora lutea, and an absence of cystic follicles [19].

In EV-induced PCOS infantile or adult rat, the ovarian content of norepinephrine (NE) is higher than in normal rats. Higher NE concentrations suggest increased activity of the sympathetic nerves and a derangement of sympathetic inputs to the ovary, two factors that contribute to the persistence of PCOS symptoms [20]. Electro-acupuncture treatment or bilateral sectioning of the SON to EV-induced PCOS rat reduces sympathetic activity and resets the animals’ estrous cycle, LH secretion, steroidogenesis and ovulation [14,15,21,22]. Unilateral sectioning of the SON restores ovulation in the innervated ovary by 80% and 26% by the denervated ovary, suggesting that in addition to an increase in sympathetic activity, other neural pathways reaching the ovaries are involved in the PCOS development [13].

According to Gerendai et al., [10,23], a multi-synaptic neural pathway between the ovary and the central nervous system (CNS) is involved in regulating ovarian functions. In the adult rat, bilateral vagotomy altered the estrous cycle [24], blocked pseudo-pregnancy induction [25], increased the number of ova shed by ovulating adult and pre-pubertal rats [26,27], and in pregnant rats resulted in lower LH basal levels, causing fetal resorption [28]. Taken together, these results suggest that the information arriving to the ovaries through the vagus nerve participates in regulating ovarian functions.

Bilateral abdominal vagotomy to pre-pubertal rats delayed the onset of puberty, and depressed E_2_ and progesterone (P_4_) response to human chorionic gonadotropin (hCG) *in vitro*[29].

Unilateral vagotomy affects spontaneous ovulation in different ways: in adult rats, sectioning the left vagus nerve resulted in lower ovulation rates, while sectioning the right vagus nerve did not have an apparent effect on ovulation rates or the number of ova shed by ovulating animals [26]. Based on these results, the researchers postulated that the neural information carried by the left vagus nerve plays a more significant role in the ovulatory process than the information carried by the right vagus nerve [26].

In pre-pubertal rats, unilateral vagotomy did not modify ovulation rates or the number of ova shed. Sectioning the right vagus nerve to 28 day old rats resulted in lower E_2_ levels and a delay of puberty onset, while sectioning the left vagus nerve had no apparent effects [27].

To our knowledge, the vagus nerve involvement in developing and regulating EV-induced PCOS rat has not been assessed. The aim of the present study was to analyze if the vagus nerve is one of the neural pathways participating in PCOS development. For this purpose we studied the effects of unilateral or bilateral vagotomy on ovarian steroidogenesis and ovulatory response in the EV-induced PCOS rats.

## Methods

All experiments were carried out in strict accordance with the Mexican Law of Animal Treatment and Protection Guidelines. The Committee of the Facultad the Estudios Superiores Zaragoza approved the experimental protocols. The study was performed using pre-pubertal female rats of the CIIZ-V strain from our own breeding stock. Animals were maintained under controlled lighting conditions (lights on from 05:00 to 19:00 h); with free access to rat chows pellets and tap water.

### Animal treatment

Ten-day old rats were injected with a single 0.1 ml corn oil (vehicle Vh) dose or 2 mg EV (Sigma Chem. Co., St. Luis, Mo. USA) dissolved in 0.1 ml corn oil. When Vh or EV injected animals reached 24 days of age they were randomly allotted to one of the following groups: 1) unilateral sectioning of the left (LSVN) or right (RSVN) vagus nerve; 2) bilateral vagotomy or; 3) sham-surgery.

Vagotomy and sham-surgery procedures were performed between 10:00 and 12:00 h. Surgeries were performed following previously described methodologies [26]. In brief, rats were anesthetized with ether and a ventral incision that included skin, muscle and peritoneum was performed. Subsequently, the liver was reflected, the esophagus exposed, and the left, right, or both vagal trunks were cut with fine forceps. Sham-surgery involved the same procedures except that the vagus trunks were not touched.

After treatment, the age of first vaginal estrous (puberty) was recorded and daily vaginal smears were taken thereafter. Vh-injected animals were sacrificed on the vaginal estrous between 90–92 days of age. EV-injected animals were sacrificed when they presented vaginal estrous at 90–92 days of age, or when they presented vaginal estrous preceeded by a proestrus smear at the similar ages.

### Autopsy procedures

Animals were sacrificed by decapitation between 10.00 AM and noon. The blood from the trunk was collected, allowed to clot, and centrifuged during 15 min at 3,000 RPM. The serum was stored at -20°C, until P_4_, T, E_2_, FSH and LH levels were measured. Following the criterion proposed by Burden y Lawrence [30], at the time of necropsy a distended stomach was considered an index of functional vagotomy.

At autopsy the oviducts were dissected and the number of ova counted with the aid of a dissecting microscope. The results were used to estimate the ovulation rate (number of ovulating animals/number in treatement group) [13].

### Ovarian morphology assessment

To assess morphology ovarian, the left ovary from each control or experimental rat was cleaned of adherent fat tissue, immersed in Bouin solution for 24 hours, dehydrated and embedded in paraffin. Ten microns-thick serial histological sections were made and stained with hematoxylin-eosin. All the sections were analyzed for the presence of corpora lutea (CL), healthy antral follicles and follicular cysts with the aid of a Nikon binocular microscope.

### Hormone measurement

E_2_ (pg/ml), T (pg/ml) and P_4_ (ng/ml) serum concentrations were measured using radioimmunoassay (RIA), with kits purchased from Diagnostic Products (Los Angeles, CA, USA). The intra- and inter-assay coefficients of variation were 8.12% and 9.28% for E_2_, 9.65% and 10.2% for T and 8.35% and 9.45% for P_4_. FSH and LH (ng/ml) levels in serum were measured by the double antibody RIA technique, employing reagents and protocols kindly supplied by the NIADDK National Pituitary Program (Bethesda, MD, USA). Intra- and inter-assay variations were in the order of 5.1% and 6.5% for LH, and 4% and 7.9% for FSH. The results are expressed in terms of NIADDK standards RP-2 FOR-FSH and LH.

### Statistical analysis

Data on P_4_, T, E_2_, FSH and LH concentrations were analyzed using Repeated Measures Analysis of Variance, followed by Dunn’s test using the GraphPad Instant 3 program. The number of ova shed by ovulating animals was analyzed using Kruskal-Wallis test, followed by Mann–Whitney *U*-test. The ovulation rate was analyzed using Chi square (Xi^2^) test. A p-value of less than 0.05 was considered significant.

## Results

### Vaginal cycle

Either Vh treatment alone or sham-surgery unilateral or bilateral vagotomy to Vh-injected rats modified the normal 4-day vaginal cycle.

The estrous vaginal smear in EV-injected rats and EV-injected rats with sham-surgery was characterized by prolonged cornified smears, followed by 2–3 days of diestrous smears. Unilateral or bilateral vagotomy did not restore the normal vaginal cycle. All the animals presented a proestrous smear followed by a day of estrous smear on the day of sacrifice.

### Ovulatory response

None of the EV injected rats showed spontaneous ovulation while all the Vh-injected animals did (0/11 vs. 12/12, p < 0.01 Fisher’s exact probability test).

**Figure 1:**
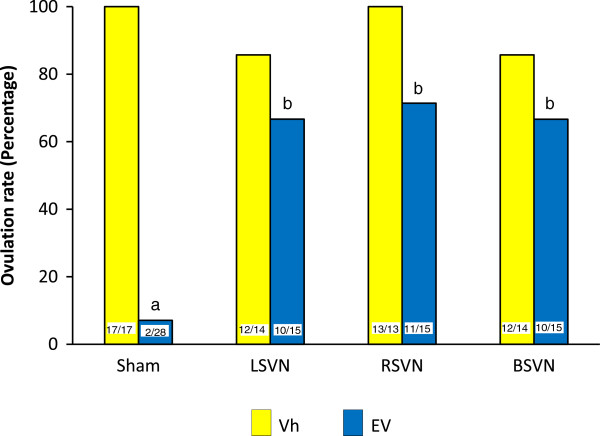
**Ovulation rate in rats with LSVN, RSVN or BSVN.** Percent of ovulating rats of rats injected with vehicle (Vh) or estradiol valerate (EV) at day 10 of life, with sham-surgery (sham) or unilateral (LSVN or RSVN) or bilateral vagotomy (BSVN) at day 24 of life, sacrificed at day 90–92 of life. The numbers at the base of the bars indicate the number of ovulating animals/number of treated animals. a p < 0.05 *vs.* Vh sham; b p < 0.05 *vs.* EV sham (Xi^2^ test).

In Vh treated-rats, sham- surgery, unilateral or bilateral vagotomy did not modify ovulation rates. In EV-injected rats 2/28 of the sham-surgery ovulated. EV-injected rats with LSVN or bilateral vagotomy showed a 65 percent ovulation rate (10/15) and the ovulation rate in EV-injected rats with RSVN was 72 percent (11/15) (Figure 1).

**Figure 2:**
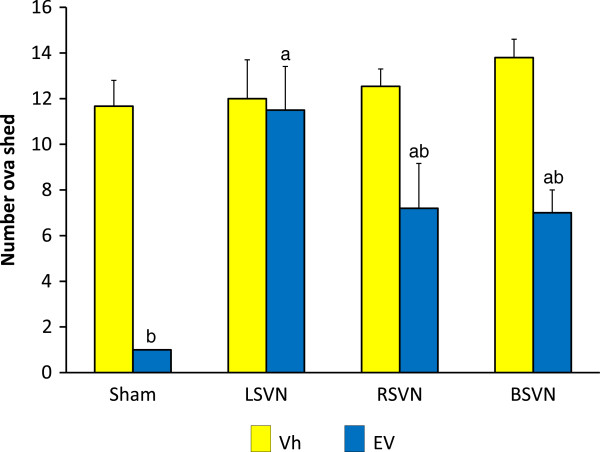
**Number ova shed in rats with LSVN, RSVN or BSVN.** Mean ± SEM of the number of ova shed in rats injected with vehicle (Vh) or estradiol valerate (EV) at day 10 of life, with sham-surgery (sham) or unilateral (LSVN or RSVN) or bilateral vagotomy (BSVN) at day 24 of life, sacrificed at day 90–92 of life. a p < 0.05 *vs.* EV sham; b p < 0.05 *vs*. paired Vh group (Kruskal-Wallis test followed by Mann–Whitney *U*-test).

The number of ova shed by Vh-injected rats with unilateral or bilateral vagotomy was similar to the Vh-injected sham-surgery group. The number of ova shed by EV treated animals with RSVN or bilateral vagotomy was lower than their respective Vh-injected groups. Compared to Vh-injected animals, LSVN to EV treated animals did not modify ovulation rates (Figure 2).

### Steroids and gonadotropin hormones levels

**Figure 3:**
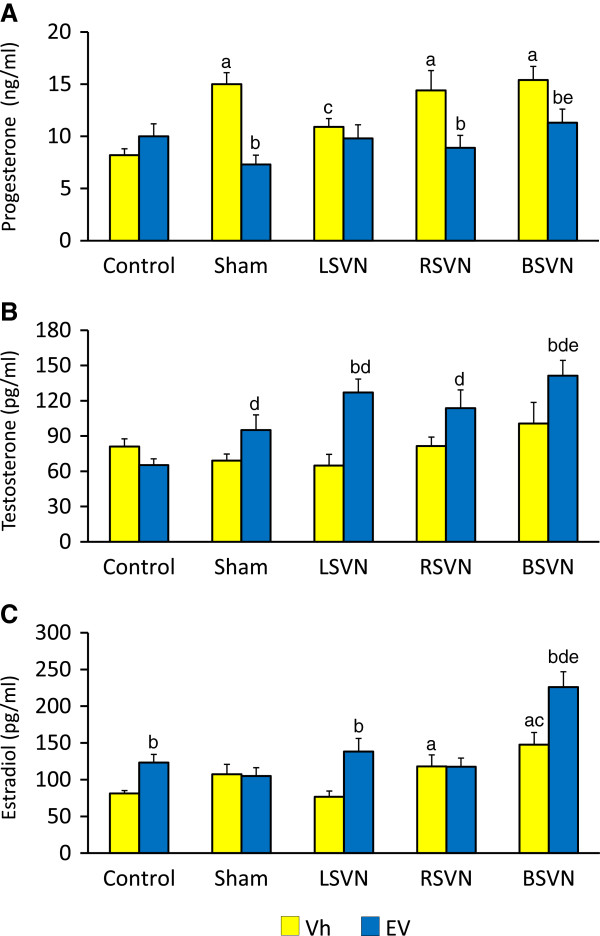
**Concentration of steroid hormones.** P_4_ (**A**), T (**B**) and E_2_ (**C**) serum levels in rats with LSVN, RSVN or BSVN. Mean ± SEM of P_4_, T and E_2_ levels in rats injected with vehicle (Vh) or estradiol valerate (EV) at day 10 of life, untouched (control), with sham-surgery (sham) or unilateral (LSVN or RSVN) or bilateral vagotomy (BSVN) at day 24 of life, sacrificed at day 90–92 of life. a p < 0.05 *vs.* Vh control group, b p < 0.05 *vs.* paired Vh group, c p < 0.05 *vs.* sham Vh group; d p < 0.05 *vs.* EV control group; e p < 0.05 *vs.* sham EV group (Repeated Measures of analysis of variance, followed by Dunn’s test).

P_4_ levels were similar in Vh-injected and EV-injected rats. Sham-surgery, RSVN or BSVN to Vh-injected rats resulted in higher P_4_ levels that Vh-injected control group. LSVN to Vh-injected rats resulted in lower P_4_ levels than in the Vh-injected sham-surgery group. Sham-surgery, RSVN or BSVN to EV-injected rats resulted in lower P_4_ levels than their respective Vh-injected group (Figure 3A).

T levels in Vh-injected and EV-injected rats were similar. Sham surgery to Vh-injected resulted in a non-significant decrease of T levels. T levels in EV-injected rats with sham-surgery, unilateral or bilateral vagotomy (LSVN, RSVN or BSVN) were higher than in EV-injected control group. T levels in EV-injected rats with BSVN were higher than in the EV-sham surgery group. Compared to the EV-sham surgery group, unilateral vagotomy (RSVN or LSVN) did not modify T levels (Figure 3B).

E_2_ levels in EV-injected rats were higher than in the Vh treatment group. E_2_ levels in the Vh-injected group with RSVN or BSVN were higher than in Vh-injected control rats. BSVN to EV-injected rats resulted in higher E_2_ levels than in EV-treated control group (Figure 3C).

EV-treated rats, with or without unilateral or bilateral vagotomy showed lower FSH levels than their respective Vh-injected groups. LH levels in EV-treated rats with unilateral or bilateral vagotomy were higher than in their respective EV-injected groups without surgical procedures (Table 1).

**Table 1:** FSH and LH serum levels in rats with LSVN, RSVN or BSVN

**Group**	**N**	**FSH**	**LH**
		**(ng/ml)**	**(ng/ml)**
**Control Vh**	12	10.4 ± 0.9	0.54 ± 0.07
**Control EV**	11	3.1 ± 0.3^**a**^	0.35 ± 0.04
**Vh + Sham**	17	7.7 ± 0.4	0.76 ± 0.04
**EV + Sham**	28	4.7 ± 0.4^**bd**^	1.18 ± 0.10^**bd**^
**Vh + LSVN**	14	7.28 ± 0.42	0.63 ± 0.05
**EV + LSVN**	15	4.10 ± 0.42^**b**^	0.94 ± 0.07^**bd**^
**Vh + RSVN**	13	6.14 ± 0.61^**ac**^	1.01 ± 0.07^**ac**^
**EV + RSVN**	15	4.0 ± 0.34^**b**^	1.14 ± 0.12^**d**^
**Vh + BSVN**	14	7.0 ± 0.6	0.82 ± 0.07^**a**^
**EV + BSVN**	15	3.2 ± 0.3^**b**^	1.04 ± 0.08^**d**^

Mean ± SEM of FSH and LH levels in animals injected with vehicle (Vh) or estradiol valerate (EV) at 10 days of life, untouched (control), with sham-surgery (sham) or unilateral (LSVN or RSVN) or bilateral vagotomy (BSVN) at day 24 of life, sacrificed at day 90–92 of life.

^a^ p < 0.05 *vs.* Vh control group, ^b^ p < 0.05 *vs.* paired Vh group, ^c^ p < 0.05 *vs.* Vh + Sham group, ^d^ p < 0.05 *vs.* EV control group (Repeated Measures of analysis of variance, followed by Dunn’s test).

### Ovarian morphology

**Figure 4:**
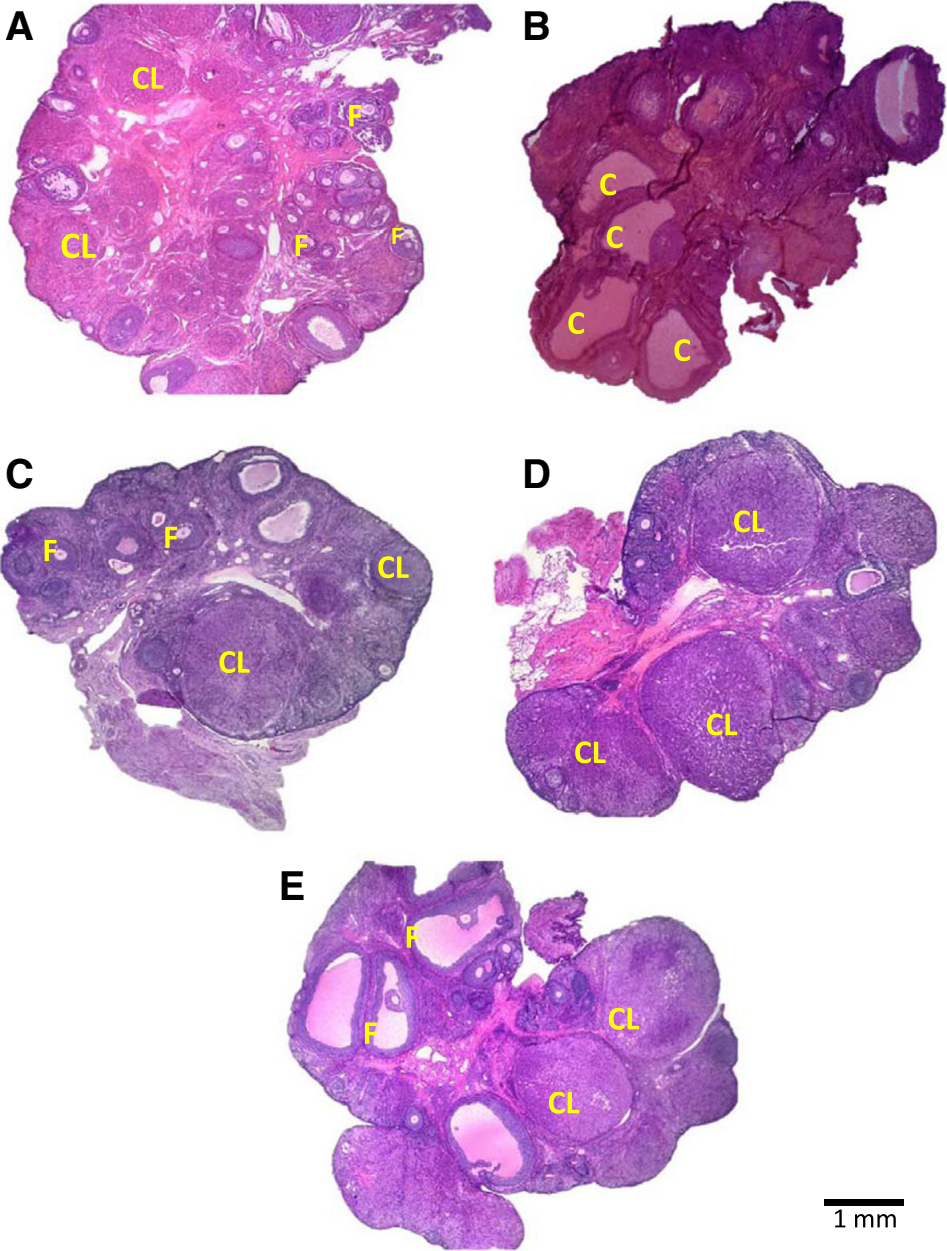
**Ovarian histology in EV-induced PCOS rat after of the LSVN, RSVN or BSVN.** Micrographs correspond to the largest section of the ovary of 10 μm thick and stained by hematoxylin-eosin, of animals sacrificed at the day with vaginal estrous smear preceeded by a day with proestrous vaginal smear. **A**, Ovary from a Vh-injected rat; **B**, polycystic ovary in EV-injected rats; **C-E**, ovary from an EV-injected rat subjected to unilateral (LSVN or RSVN) or bilateral vagotomy (BSVN) at day 24 of life, sacrificed at day 90–92 of life. F, normal follicle; CL, corpora lutea; C, follicular cystic.

Figure 4 shows an ovary of a control animal sacrificed on estrous day, where several fresh corpora lutea, as well as some antral follicles can be observed (A). The histological analysis of the ovary of rats with EV- induced PCOS revealed the presence of cystic follicles and no corpora lutes (B). LSVN (C), RSVN (D) and BSVN (E) treatment to EV-induced PCOS rats changed the morphological aspects of the ovaries. Numerous fresh corpora lutea were readily apparent as well as marked attenuation of the cystic condition.

## Discussion

The results obtained in the present study suggest that the neural information carried by the vagus nerve plays a role in the mechanisms participating in the regulation of development and maintenance of PCOS.

The development of ovarian patho-physiologic conditions, such as the PCOS, result from alterations in the neuroendocrine axis regulating ovarian function. Several hypothesis have been proposed to explain the etiology of the PCOS, including failings in the pulsatile secretion of gonadotropin release hormone (GnRH) and the resulting deficiencies in ovarian sex steroid synthesis or metabolism [7]; the hyper-activation of the sympathetic fibers arriving to the ovary via the SON [21,31]; and kisspetin related mechanisms [32]. Injecting LHRH to EV-induced PCOS rat induces ovulation, suggesting that alterations to LHRH secretion by the hypothalamus are one of the main conditions that favor PCOS development and maintenance on the female reproductive system [17,18].

The ovarian innervation plays a role modulating the reactivity of the ovaries to gonadotropins. The NE and vasoactive intestinal peptide (VIP) fibers carried by the SON stimulate FSH receptor synthesis [33]. In EV-induced PCOS rat, the bilateral sectioning of the SON [13-15] or the bilateral electro-acupuncture treatment at the T12-L2 segments level [21] result in spontaneous ovulation and lower ovarian NE levels. Despite a drop of NE levels in the denervated ovary, unilateral sectioning of the SON restored ovulation by the innervated ovary; suggesting that lower NE content are not the only factor acting to restore ovulation [13]. Then, it is possible that PCOS onset is triggered by the hyperactivity of the sympathetic ovarian innervation and other non-adrenergic factors, such as VIP [34].

Gerendai et al., [10] suggested that the ovaries and CNS are linked by a neural loop, where the ovaries receive neural information from the CNS via the SON, OPN and vagus nerve. The ovaries send neural information via the SON and celiac-superior mesenteric ganglia (CSMG) and sensitive via by the vagus nerve [35-37].

Bilateral vagotomy to adult [26] and pre-pubertal rats [27], results in a higher ova shed numbers by ovulating animals. On the other hand, unilateral sectioning of the SON results in lower numbers of ova shed by denervated ovary [37]. Present results suggest that in EV-induced PCOS rat the participation of the vagus nerve in regulation ovarian patho-physiology is different than the participation of the SON.

According to Evans and Murray [38] and Agostoni et al., [39], 85–90 percent of vagus nerve fibers carry information from the peripheral organs to the CNS. On the other hand, the SON carries neural information from the CSMG to and from the ovaries [35,36]. In EV-induced PCOS rat, the most striking differences resulting from unilateral sectioning the vagus nerve or the SON are on spontaneous ovulation and hormone secretion changes. Unilateral vagotomy to EV-induced PCOS rat restored ovulation in both ovaries, while unilateral sectioning of the SON restored ovulation only in the innervated ovary. Such difference does not seem related to gonadotropins concentration since EV-induced PCOS rats with unilateral vagotomy had lower FSH levels than Vh injected rats. In turn, unilateral sectioning of the SON in EV-induced PCOS rats resulted in FSH levels similar to the control group [13]. The different types of neural information carried by the vagus nerve and the SON could explain the differences observed in gonadotropin levels in EV-induced PCOS rats submitted to unilateral denervation.

The main Hypothalamic-Pituitary-Adrenal (HPA) axis regulators are the corticotropin-releasing hormone (CRH) and the vassopresine hormone. Both stimulate pituitary adrenocorticotropic hormone (ACTH) secretion and the subsequent secretion of cortisol and P_4_ by the adrenal cortex. The stress activation of the HPA axis inhibits female reproductive function [40]. Sham-surgery in Vh-injected rats resulted in higher P_4_ levels, suggesting that the increase in P_4_ levels resulting from the HPA axis activation also participate in inhibiting ovarian functions.

P_4_ levels increases in Vh-injected groups with right or bilateral vagotomy arise from the effects of the sham-surgery, similar to other stressfull situations effects [41]. The adrenals receive vagal innervation directly and by the adrenal nerve originating in the celiac ganglia [42]. Present results suggest that the increase in E_2_ levels resulting from injecting EV reduced the ability of the fresh corpora lutea and/or the adrenals ability to secrete P_4_.

T levels in EV-induced rats PCOS have been described as higher [13], lower [15,16], and similar to those of control groups [14,17]. Such discrepancies have been explained by the rapid conversion of T to E_2_ at the ovary and/or its periphery [15]. In the present study, T levels in EV-induced PCOS rats were similar to control animals. E_2_ levels in EV-induced PCOS rats were three times higher than in control rats sacrificed at 13.00 of proestrus [43]. These higher E_2_ levels are relatively similar to those observed in previous EV-induced PCOS rat studies [13,15,18].

The higher T and E_2_ levels observed in EV-induced PCOS rat with bilateral vagotomy suggest that the vagus nerve plays an inhibitory role on hormone secretion. Then, in EV-induced PCOS rats, the role played by the vagus nerve on T and E_2_ secretion is opposite to the role played by the SON, since the bilateral sectioning of the SON decreases T and E_2_ levels [13].

Dissen et al., [44] suggested that in humans and rodents the overproduction of ovarian nerve growth factor (NGF) is a component of polycystic ovarian morphology. Persistently higher LH levels in plasma are required for the morphological abnormalities to appear, and under normal conditions, the ovulatory process is facilitated by the ovarian NGF acting via high affinity tyrosine receptor kinase A [44]. However, an excess of ovarian NGF initiates pathological changes in both endocrine and non-endocrine tissues [45]. Therefore, it is possible that bilateral vagotomy treatment disrupted the afferent network involved in the ovarian feedback of GnRH/LH secretion as proposed by Gerendai et al., [10,23].

According to Lara et al., [31], the hyper-activation of the ovarian sympathetic input resulting from EV treatment is related to an overproduction of ovarian NGF and its low affinity receptor in the ovary. Although EV-induced follicular cysts are first detected around 60 days after EV treatment [31,46], activation of the ovaries’ sympathetic innervation occurs at least a month before the formation of follicular cysts [20]. In turn, increases in p75 NGFR synthesis occur as early as 15 days after EV treatment and is shortly followed by increases in NGF synthesis [31]. This suggests that the activation of this ligand/receptor module is an early event in the process by which EV treatment disrupts ovarian function. NGF increased in the sympathetic neurons projecting to the ovary are likely to play a significant role in enhancing the sympathetic outflow to the ovary in EV-treated rats [31]. Then, it is possible that the vagus nerve participates in regulating NGF release by the sympathetic neurons that origin in the CSMG.

Very little is known about the mechanism by which vagotomy alters ovarian function. It has been proposed that the vagus nerve carries sensory fibers that influence gonadotropin secretion by acting on the hypothalamus [28] and modifying the effects of gonadotropin on the ovary [26]. Present results suggest that this regulation depends of physiological environment of the animal. Based on the results presented herein, and according to Berthoud and Powley [47], there is an apparent communication between the sympathetic and parasympathetic fibers at the CSMG level. We suggest that the vagus nerve serves as a communication channel between the ovaries and that, in rats, this channel is closely related to the development and persistence of EV-induced PCOS. The spontaneous ovulation observed in EV-induced PCOS rat with unilateral or bilateral vagotomy is evidence that the neural information carried by the vagus nerve participates, directly or indirectly, in the regulation of the development and persistence of the PCOS.

Since in the PCOS affected animals the mechanisms regulating GnRH secretion are altered [17,18], and these alterations may be modified by vagotomy procedures, present results suggest that neural signal originating from each ovary would indicate the physiological conditions of the ovaries to the CNS, which in turn participates in the regulation of GnRH secretion [37].

## Conclusions

The results suggest that in the EV-induced PCOS rats the CSMG is a neural regulation center where the vagus nerve acts on the neurons originating the SON.

## Competing interests

The authors declare that they have no competing interests.

## Authors’ contributions

RL, LM and RD planned the experiments. LM, RL, DH, CM and RD devised the study and participated in the discussion of the results. RC and MC participated in performing the RIA’s to measure the different hormones levels. All authors read approved the final manuscript.
